# Smchd1-Dependent and -Independent Pathways Determine Developmental Dynamics of CpG Island Methylation on the Inactive X Chromosome

**DOI:** 10.1016/j.devcel.2012.06.011

**Published:** 2012-08-14

**Authors:** Anne-Valerie Gendrel, Anwyn Apedaile, Heather Coker, Ausma Termanis, Ilona Zvetkova, Jonathan Godwin, Y. Amy Tang, Derek Huntley, Giovanni Montana, Steven Taylor, Eleni Giannoulatou, Edith Heard, Irina Stancheva, Neil Brockdorff

**Affiliations:** 1 Department of Biochemistry, University of Oxford, Oxford OX1 2JD, UK; 2 Computational Biology Research Group, University of Oxford, Oxford OX1 2JD, UK; 3 MRC Clinical Sciences Centre, Faculty of Medicine ICSTM, Hammersmith Hospital, Du Cane Road, London W12 0HS, UK; 4 Wellcome Trust Centre for Cell Biology, University of Edinburgh, Michael Swann Building, Mayfield Road, Edinburgh EH9 3JR, UK; 5 Centre for Bioinformatics, Imperial College London, London SW7 2AZ, UK; 6 Department of Mathematics, Imperial College London, London SW7 2AZ, UK; 7 Genetics and Developmental Biology Unit, Institut Curie, CNRS UMR3215, INSERM U934, 75005 Paris, France

## Abstract

X chromosome inactivation involves multiple levels of chromatin modification, established progressively and in a stepwise manner during early development. The chromosomal protein Smchd1 was recently shown to play an important role in DNA methylation of CpG islands (CGIs), a late step in the X inactivation pathway that is required for long-term maintenance of gene silencing. Here we show that inactive X chromosome (Xi) CGI methylation can occur via either Smchd1-dependent or -independent pathways. Smchd1-dependent CGI methylation, the primary pathway, is acquired gradually over an extended period, whereas Smchd1-independent CGI methylation occurs rapidly after the onset of X inactivation. The de novo methyltransferase Dnmt3b is required for methylation of both classes of CGI, whereas Dnmt3a and Dnmt3L are dispensable. Xi CGIs methylated by these distinct pathways differ with respect to their sequence characteristics and immediate chromosomal environment. We discuss the implications of these results for understanding CGI methylation during development.

## Graphical abstract

**Graphical Abstract:**

**Figure:**
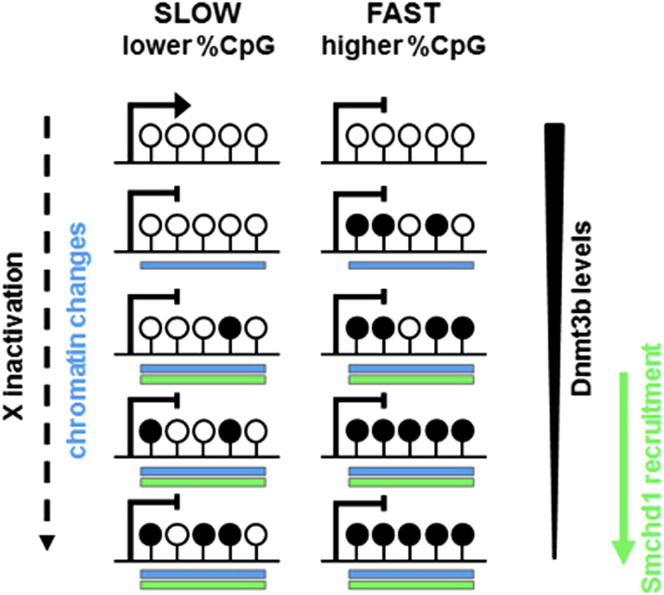

**Highlights:**

► Xi CGIs exhibit either fast or slow DNA methylation kinetics ► Xi CGI methylation requires Dnmt3b but not Dnmt3a or Dnmt3L ► Recruitment of the chromosomal protein Smchd1 is a late step in X inactivation ► Slow, but not fast, Xi CGI methylation requires the chromosomal protein Smchd1

## Teaser

What mechanisms contribute to developmental gene regulation and long-term silencing? Gendrel et al. uncover on the inactive X chromosome parallel pathways of slow and fast CpG-island methylation kinetics associated with different chromosomal contexts. Only slow methylation needs the chromosomal protein Smchd1, but both pathways require the de novo methyltransferase Dnmt3b.

## Introduction

DNA methylation in higher organisms functions to repress transcription of genes, cryptic promoters, and repetitive sequences (Bird and Wolffe, 1999; Skene et al., 2010; Walsh et al., 1998). In mouse, DNA methylation patterns undergo dynamic changes during normal development. Methylation inherited from either the maternal or paternal germline is largely erased during early embryo development, and subsequently de novo methyltransferases Dnmt3a and Dnmt3b together establish appropriate methylation patterns that are then heritably maintained through cell division by the action of the maintenance DNA methyltransferase, Dnmt1. DNA methylation is required for normal development, as evidenced by mutations affecting the de novo and maintenance methyltransferases (reviewed in Hermann et al., 2004).

Methylation occurs through addition of a methyl group to the 5′ position of a cytosine residue, and in mammals this happens predominantly on both strands of the palindromic cytosine-guanine (CpG) dinucleotide. CpG dinucleotides are generally distributed at low density and are highly methylated (Bird et al., 1985; McClelland and Ivarie, 1982). However, a proportion of CpGs are clustered in ∼1000-bp-long domains known as CpG islands (CGIs). CGIs are frequently associated with the promoter region of genes and are generally methylation free, regardless of the transcription status of the associated gene (Bird, 2002; Bird et al., 1985).

Developmentally regulated methylation of CGIs occurs in specific circumstances and is usually linked to silencing of associated genes. Thus, the CGIs of parentally imprinted genes are frequently methylated either in the germline or during early development (Edwards and Ferguson-Smith, 2007; Neumann and Barlow, 1996). CGIs on the inactive X chromosome are methylated during normal development (Lock et al., 1987; Norris et al., 1991), as is a subset of developmentally regulated genes that are methylated in a tissue or developmental stage-specific manner (Borgel et al., 2010; Fazzari and Greally, 2004; Illingworth et al., 2008; Mohn et al., 2008; Oda et al., 2006; Smith et al., 2012; Strichman-Almashanu et al., 2002). Finally, certain CGIs become methylated as a consequence of cancer, aging, or prolonged passaging in tissue culture (Antequera et al., 1990; Issa et al., 1994; Jones et al., 1990).

The mechanism by which CGIs are normally protected from de novo DNA methylation is poorly understood. The fact that CGIs on the X chromosome and at imprinted loci can be either methylated or unmethylated demonstrates that there is no intrinsic DNA sequence characteristic that confers this protection. One idea, supported by studies on the APRT promoter, is that binding of ubiquitous transcription factors precludes de novo DNA methyltransferase association (Brandeis et al., 1994; Macleod et al., 1994). More recent evidence suggests that histone H3 lysine 4 methylation in promoter regions can block direct interaction of de novo DNA methyltransferases with the chromatin template (Ooi et al., 2007). Possibly related to this, chromatin-modifying factors containing a CXXC domain that confers specific binding to unmethylated CpG were recently shown to localize widely to CGIs (Blackledge et al., 2010; Thomson et al., 2010).

X inactivation is the dosage compensation mechanism used by mammals to equalize levels of X-linked genes in females relative to males (for recent reviews, see Augui et al., 2011; Senner and Brockdorff, 2009; Wutz, 2011). The process is triggered in early development by the expression and chromosome-wide accumulation of the noncoding RNA, Xist. Chromosome coating by Xist RNA sets in play a cascade of chromatin modifications, culminating in stable, long-term silencing of the majority of X-linked genes. Inactive X (Xi) chromatin modifications include specific histone modification signatures, enrichment of variant histones, recruitment of proteins that influence chromosome structure, and DNA methylation of promoter-associated CGIs. The mechanism by which Xist RNA initiates these changes, and the interplay and interdependence of the different modifications are poorly understood.

Previous studies indicated that DNA methylation is a late step in the X inactivation process and is likely important for the long-term maintenance of X inactivation (Grant et al., 1992; Keohane et al., 1996; Lock et al., 1987; Singer-Sam et al., 1990), a view that is supported by genetic studies (Blewitt et al., 2008; Sado et al., 2000). In this study, we provide a detailed characterization of DNA methylation in X inactivation. We demonstrate that the de novo methyltransferase Dnmt3b is specifically required for the methylation of CGIs on Xi. An analysis of the developmental dynamics of Xi CGI methylation reveals two independent modes. In a large proportion of CGIs, DNA methylation accumulates slowly throughout ontogeny, and, as reported previously (Blewitt et al., 2008), this is dependent on recruitment of the chromosomal protein Smchd1 to Xi. However, a subset of CGIs show fast methylation kinetics that in many cases occurs independently of Smchd1. Methylation kinetics and Smchd1 dependence are linked to the inherent characteristics of CGIs, their immediate chromosomal environment, and the expression profile of associated genes.

## Results

### CGI Methylation Dynamics on Xi during XX Embryonic Stem Cell Differentiation

**Figure 1:**
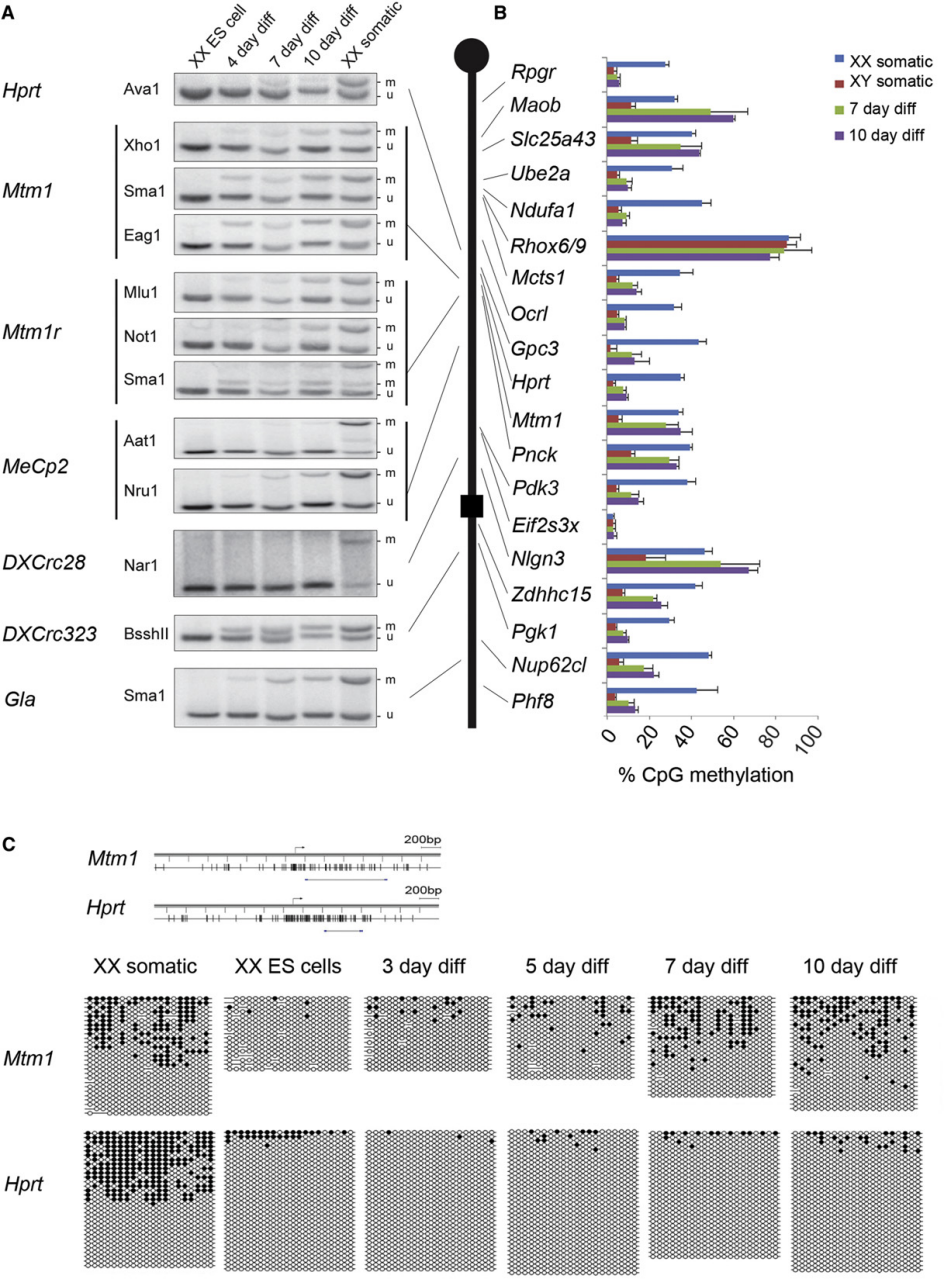
Fast and Slow Methylation Dynamics of Xi CGIs in Differentiating XX ES Cells (A) Southern analysis of MSRE digests using indicated restriction enzymes. Presence of methylated (m) and unmethylated (u) sites were determined in XX ES cells, in XX ES cells differentiated (diff) for 4, 7, and 10 days, and in XX somatic controls (adult kidney). Multiple sites within the same CGI are linked with a vertical line. The schematic indicates the chromosomal location of CGI-associated loci. (B) Sequenom EpiTyper analysis of CGIs associated with genes along the length of the X chromosome, as indicated on the schematic. Average CpG methylation levels were determined for XX and XY somatic tissue (adult kidney) and XX ES cells differentiated (diff) for the times shown. Error bars indicate positive SD values from three independent experiments. (C) Bisulfite DNA sequencing analysis of *Mtm1* and *Hprt* CGIs in XX somatic cells (adult kidney), XX ES cells, and ES cells differentiated (diff) for the times shown. Schematics illustrate the regions analyzed by bisulfite sequencing (horizontal line below map), transcription start site (arrows), and CpG density (vertical lines). Bottom: Each line represents methylation on an individual DNA strand determined by sequencing subcloned PCR product from bisulfite-treated genomic DNA. Methylated and unmethylated CpGs are indicated with closed and open circles, respectively. Ambivalent sequence reads are shown as gaps. See also Table S1.

Differentiating XX embryonic stem (ES) cells provide an in vitro model that recapitulates the dynamic changes that occur during initiation and establishment of X inactivation in embryos (Chaumeil et al., 2004). We used this model to analyze methylation changes at CpG sites within several X-linked CGIs at time points during XX ES cell differentiation, initially using methylation-sensitive restriction enzyme (MSRE) analysis. We identified MSRE sites that were unmethylated in undifferentiated XX ES cells and showed methylation levels of ∼50% in XX somatic cells (consistent with methylation on the inactive X allele) and then analyzed the methylation of these sites during the course of XX ES cell differentiation (embryoid body formation) for up to 10 days (Figure 1A). MSRE sites in the *Hprt*, *MeCp2*, and *DXCrc28* CGIs showed little or no DNA methylation over the entire differentiation time course. For *Hprt*, this was known to be the case from an earlier study (Lock et al., 1987). Other sites, however, acquired significant methylation levels (*Mtm1* and *DXCrc323*) or were methylated at an intermediate level (*Mtm1r* and *Gla*) over the 10 day time course.

We substantiated these initial findings for a number of CGIs across the length of the X chromosome using Sequenom EpiTyper analysis of bisulfite-treated samples (Ehrich et al., 2005). Averaged data for sites within a given CGI are shown in Figure 1B, and data for individual CpG sites are provided in Table S1 (available online). The *Rhox6/9* CGI, which is known to be methylated on both the active (Xa) and inactive (Xi) chromosomes (Oda et al., 2006), and the CGI associated with Eif2s3x, a gene that escapes X inactivation (Yang et al., 2010), served as controls. Similar to the MSRE analysis, methylation dynamics at individual CGIs were found to vary, showing fast (*Maob*, *Slc25a43*, *Mtm1*, *Pnck*, and *Nlgn3*), intermediate (*Gpc3*, *Pdk3*, *Zdhhc15*, *Nup62cl*, and *Phf8*), or slow (*Rpgr*, *Ndufa1*, *Mcts1*, *Ocrl*, *Hprt*, and *Pgk1*) kinetics.

We carried out a direct bisulfite sequencing analysis to further validate representative fast- and slow-methylating CGIs (*Mtm1* and *Hprt*; Figure 1C). In XX somatic cell samples, ∼50% of the strands show significant methylation (presumed Xi allele), and the remainder are largely unmethylated (presumed Xa allele). In XX ES cells, where both X chromosomes are active, *Hprt* and *Mtm1* CGIs are hypomethylated. During ES cell differentiation, both CGIs acquired methylation gradually but at very different rates. For *Mtm1*, DNA methylation levels increased rapidly across the region tested from 1.3% in the undifferentiated ES cells to 5.1%, 9.4%, 22.1%, and 20.5% at days 3, 5, 7, and 10, respectively. In marked contrast, *Hprt* methylation levels remained low, at 0.2%, 2.07%, 2.18%, and 3.29% at the same time points.

**Figure 2:**
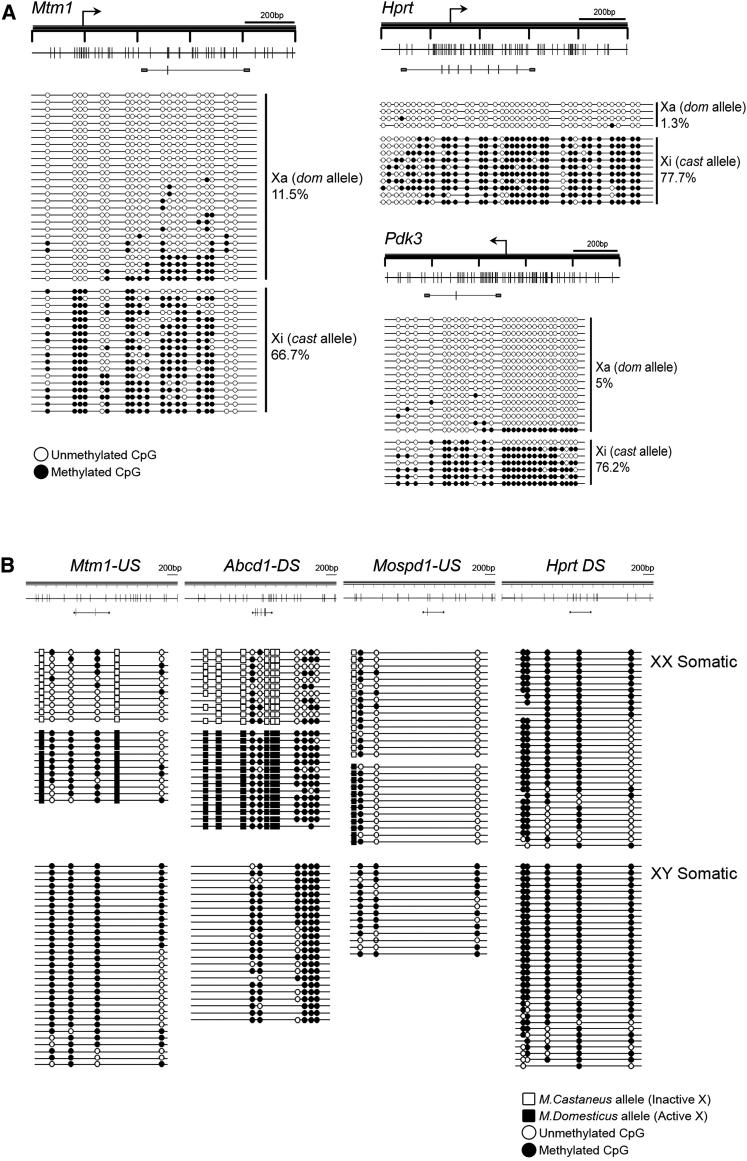
Allelic CpG Methylation Patterns on Xa and Xi (A) Bisulfite DNA sequencing assays of CGIs of *Mtm1*, *Hprt*, and *Pdk3* CGIs in T16H/Cast XX somatic cells. Schematics illustrate the regions analyzed by bisulfite sequencing. SNPs between *M. domesticus* (dom) Xa and *M. castaneus* (cast) Xi alleles were used to assign the origin of individual strands. Average % methylation of Xa and Xi alleles is indicated. (B) Bisulfite DNA sequencing analysis of CpG sites upstream (US) and downstream (DS) of X-linked genes as indicated in T16H/Cast XX somatic cells and an XY somatic MEF cell line (E4.22.5). Schematics illustrate maps of four non-CGI regions indicating the region amplified by PCR (horizontal line below map) and CpG sites (vertical lines). *M. domesticus* and *M. castaneus* SNPs (open and closed square boxes, respectively) are indicated together with bisulfite sequencing patterns.

We verified confinement of CpG methylation to the Xi allele for selected genes (*Hprt*, *Mtm1*, and *Pdk3*) by carrying out bisulfite sequencing analysis of DNA from an XX fibroblast cell line, T16H/Cast (Gómez and Brockdorff, 2004), in which there is nonrandom X inactivation. SNPs between *Mus domesticus* and *M. castaneus* X chromosomes were used to distinguish between Xi and Xa alleles (Figure 2A).

Previous studies have found that methylation of CGIs on Xi is accompanied by loss of methylation at intronic/intergenic CpGs, and that overall CpG methylation is lower on Xi than on Xa (Hellman and Chess, 2007; Lock et al., 1986; Weber et al., 2005). To confirm this, we analyzed the methylation of intergenic/intronic CpGs in T16H/Cast XX and an XY cell line, using either SNPs or differences between XX and XY cells to distinguish between Xi and Xa methylation (Figure 2B). We analyzed a total of four regions, three of which had informative polymorphisms. Two of the polymorphic regions (those linked to the *Mtm1* and *Abcd1* loci, respectively) were found to be hypomethylated on the Xi allele but not on the Xa allele, i.e., the converse of what was observed for X-linked CGIs. The polymorphic region upstream of the *Mospd1* locus was hypomethylated on both Xa and Xi. Methylation of *Mopsd1* sites was seen in the XY somatic cell line, likely due to cell-line-specific effects of long-term tissue culture. A single CpG site in the nonpolymorphic region located downstream of *Hprt* was methylated in male DNA, but in only around half of the strands in female DNA, indicating that here also there is Xi-specific hypomethylation. These results substantiate that CGI methylation on Xi is often accompanied by hypomethylation of intergenic/intronic sequences.

### Xi CGI Methylation Dynamics In Vivo

**Figure 3:**
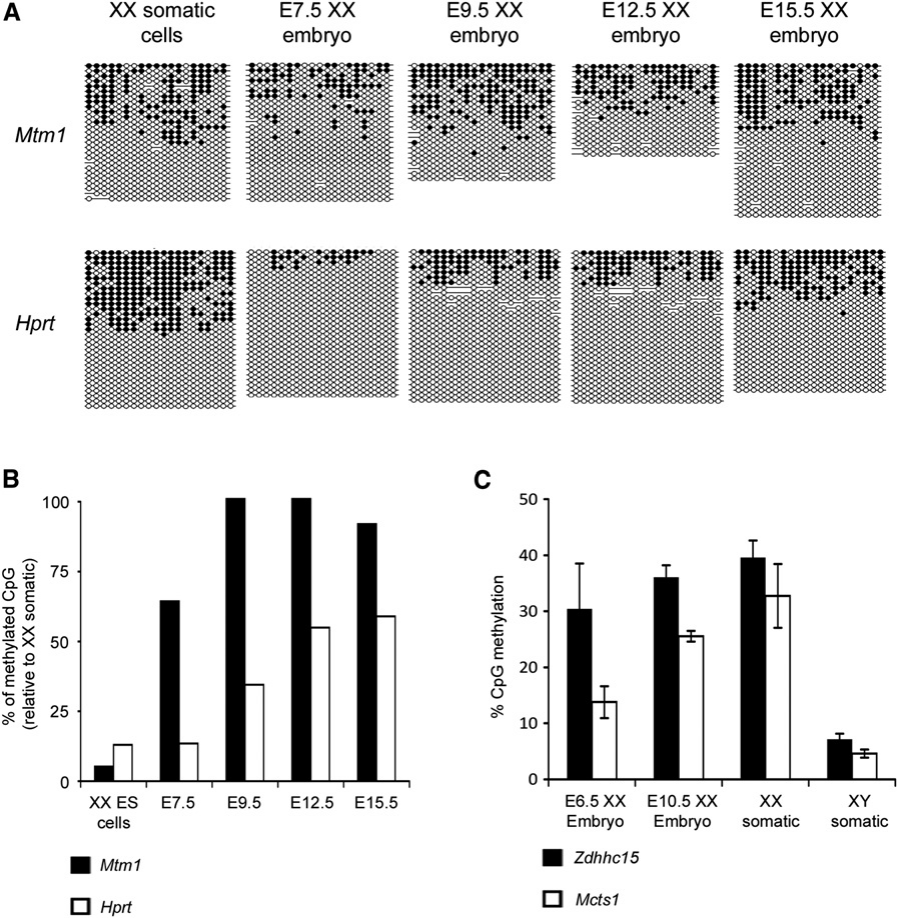
Xi CGI Methylation Dynamics In Vivo (A) Analysis of *Mtm1* and *Hprt* CGIs in DNA from pooled XX embryos isolated at developmental stages as indicated by bisulfite DNA sequencing. The regions analyzed are as shown in Figure 1, and XX somatic cell data from Figure 1 are included for illustrative purposes. (B) Graphical representation of data in (A) illustrating methylation at *Mtm1* and *Hprt* as a percentage of methylation levels in XX somatic cells. (C) Sequenom EpiTyper analysis of methylation at CGIs associated with *Zdhhc15* and *Mcts1* in E6.5 and E10.5 XX embryos and somatic cell controls (adult kidney). The % CpG methylation is averaged for sites across the region analyzed. Error bars indicate variation between individual embryos (n = 3) or adult tissue DNA samples (n = 3). See also Table S1.

We went on to test whether Xi CGI methylation dynamics observed in differentiating XX ES cells are recapitulated in XX embryos undergoing random X inactivation. Initially, we analyzed the methylation levels of the *Mtm1* and *Hprt* CGIs that in differentiating XX ES cells show fast and slow methylation kinetics, respectively. X inactivation initiates around E5.5–E6.5, and we therefore analyzed subsequent developmental time points between E7.5 and E15.5 (Figures 3A and 3B). In the *Mtm1* CGI, methylation accumulated to maximal levels, equivalent to that observed in female somatic cells, by E9.5. In contrast, methylation levels in *Hprt* accumulated slowly and were lower than in XX somatic cells even at E15.5.

To extend these findings, we used Sequenom EpiTyper analysis of bisulfite-treated DNA to analyze CGIs associated with the *Zdhhc15* and *Mcts1* loci, identified as intermediate and slow methylating islands respectively, in E6.5 and E10.5 XX embryos (Figure 1B; Table S1). Consistent with data for differentiating XX ES cells, the methylation rate of *Zdhhc15* in vivo was significantly faster than that observed for *Mcts1* (Figure 3C). These results demonstrate that the fast, intermediate, and slow CGI methylation kinetics observed in differentiating XX ES cells reflect real differences in CGI methylation that occur during the X inactivation process in vivo.

### Chromosome-wide Analysis of Xi CGI Methylation Dynamics

**Figure 4:**
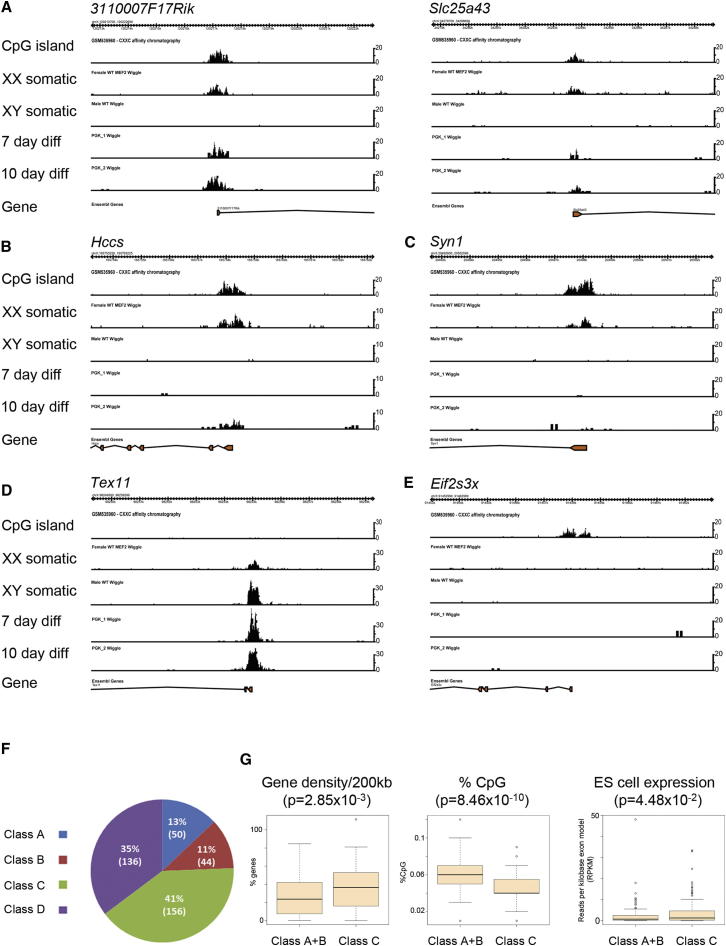
Chromosome-wide Analysis of CGI Methylation Dynamics in Differentiating XX ES Cells (A–E) Browser screenshots of 10 kb regions illustrating examples of major classes of Xi CGI methylation patterns detected in MBD-seq analysis of XX and XY somatic cells (MEF cell lines) and XX ES cells differentiated (diff) as indicated. The y axis indicates number of reads. The CGI plots are from a previously published CAP-seq analysis of DNA from mouse cerebellum (Illingworth et al., 2010). (F) Pie chart illustrating the proportion of major CGI methylation patterns as defined in the text. (G) Illustrative examples of significant differences in gene density, % CpG, and ES cell expression level of associated genes from a comparison of class A+B genes with class C genes. See also Tables S2 and S3.

To determine the proportion of Xi CGIs in different dynamic classes, we performed whole-genome analysis of CGI methylation using high-throughput (HT) sequencing of highly methylated DNA sequences purified by methyl binding domain sequencing (MBD-seq) (Cross, 2002). CGI methylation profiles were determined for XX and XY somatic cells and for XX ES cells that had differentiated for either 7 or 10 days. To functionally define the CGI location, we made use of a recently published data set obtained by CXXC affinity purification chromatography coupled with HT sequencing (CAP-seq) of DNA from mouse cerebellum (Illingworth et al., 2010). Examples are shown in Figures 4A–4E. We were able to identify three major classes of CGI with Xi-specific methylation: those that acquire significant methylation at 7 days and/or 10 days of differentiation (Figure 4A, *311000F17Rik* and *Slc25a43*), those for which significant methylation can be detected at day 10 of differentiation (Figure 4B, *Hccs*), and those that remain unmethylated at both time points (Figure 4C, *Syn1*). Additionally, we identified loci where promoter methylation occurs in both males and females, indicating methylation on Xa and Xi (Figure 4D, *Tex11*), and loci with negligible methylation of CGIs in XX somatic cells, notably those associated with genes that escape X inactivation (Figure 4E, *Eif2s3x*).

We used a bioinformatic analysis of the data sets to estimate the proportion of Xi CGIs that showed different methylation kinetics. We first excluded CGIs in which we could detect similar levels of methylation in XY and XX somatic cells, and also those that were unmethylated in all cell types. Thus, we identified a total of 386 CGIs with Xi-specific methylation. The vast majority of these CGIs were associated with the promoter region of known genes (Table S2). We then categorized the methylation dynamics of the CGIs as fast methylating (class A), methylated at day 10 of differentiation only (class B), or slow methylating (class C; see Supplemental Experimental Procedures). CGIs for which methylation dynamics could not be assigned to any of these classes were considered as a separate category (class D). On this basis, we determined that classes A, B, and C comprise 11%, 13%, and 41% of all CGIs, respectively (Figure 4F), which indicates that Xi CGIs that undergo slow methylation kinetics are the predominant category.

Several studies have identified characteristics of CGIs or their immediate chromosome environment that may contribute to a propensity to acquire CpG methylation (Bock et al., 2006; Illingworth et al., 2008; Illingworth et al., 2010; Jia et al., 2007). To investigate whether any of these parameters could play a role in determining differential methylation dynamics on Xi, we carried out pairwise comparisons for categories A–D using features that probe for association with local chromosome environment (gene density, LINE1 repeat density, and distance from the *Xist* locus), CGI characteristics (length of CGI, CpG density, GC content, CpG observed/expected ratio, and twist/stacking energy), and finally expression of associated genes (RNA expression in ES cells and Ring1B Polycomb repressor targets). A full list of the parameters tested and the results obtained is provided in Table S3. We found that class A and B CGIs (both of which are methylated in differentiating XX ES cells) differ significantly from class C CGIs (which are unmethylated in XX ES cells even at day 10) in terms of CGI characteristics; in particular, the former have a higher CpG density and GC content. In addition, we found that class A CGIs are located in domains with lower gene density and are associated with genes that have relatively low levels of expression in ES cells. Consistent with the latter, X-linked ES cell Polycomb target genes were largely present within class A. In a simplified analysis, we observed similar associations by comparing CGIs in classes A and B with CGIs in class C (Table S3). Specific examples illustrating the association of class C with high gene density, low CpG density, and higher levels of expression in ES cells are shown in Figure 4G.

Class B genes additionally showed a distinct distribution on the X chromosome, being located closer to the *Xist* locus relative to other classes. Comparisons of class D with other classes also revealed similarities and differences. The possible relevance of such associations, however, is unclear given that these loci could not be clearly assigned to any of the defined dynamic groups.

### A Role for Dnmt3b in De Novo Methylation of Xi CGIs

**Figure 5:**
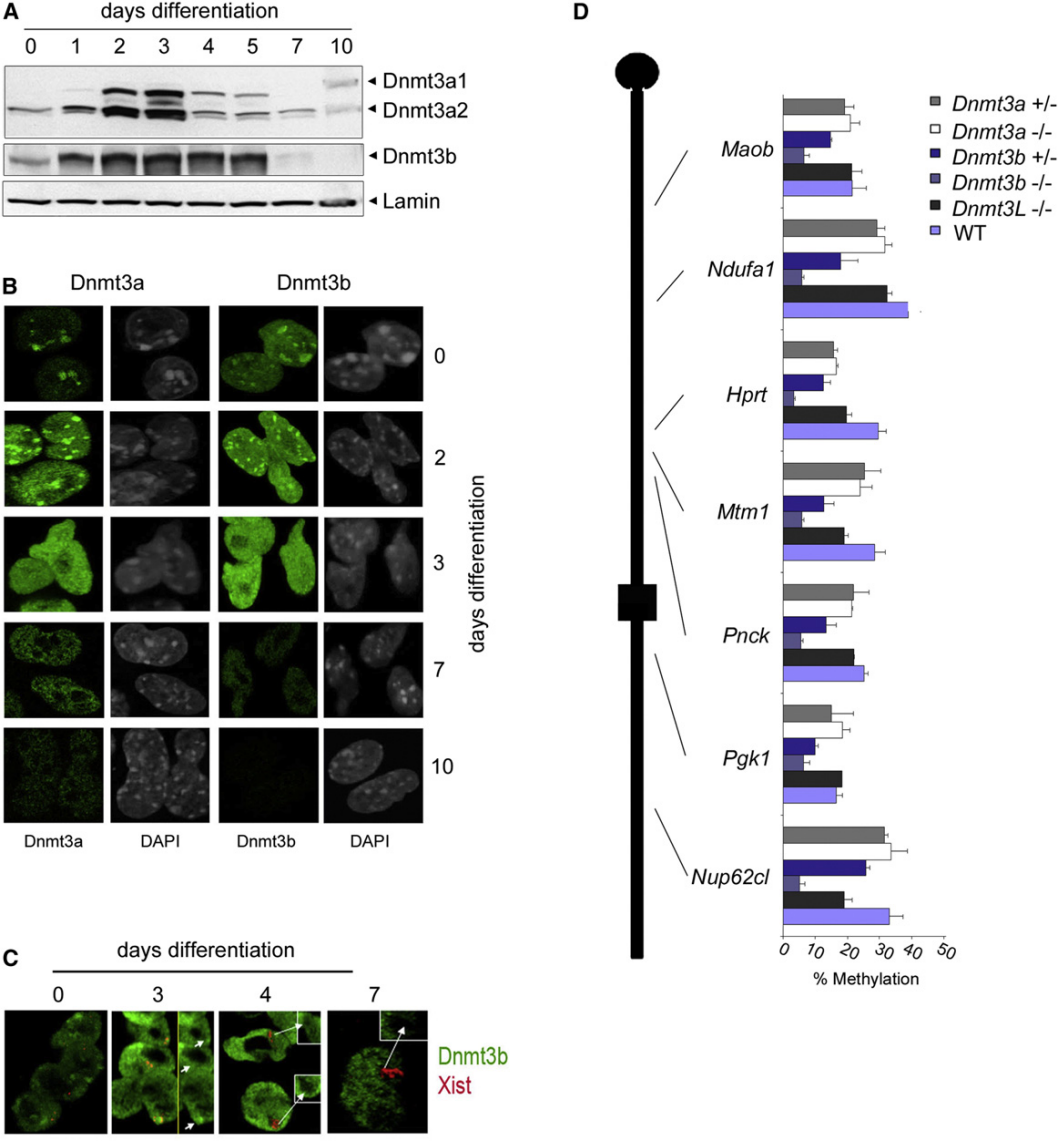
Dnmt3b Is Required for Xi CGI Methylation (A) Western blot analysis illustrating levels of Dnmt3a isoforms a1 and a2, and the major Dnmt3b isoform during differentiation of XX ES cells in vitro. Detection of Lamin B was used as a loading control. (B) Examples of IF for Dnmt3a and Dnmt3b, as indicated, in XX ES cells and following in vitro differentiation for the times shown. DNA was counterstained with DAPI. Staining patterns reveal overall nuclear localization of Dnmt3a/b and, at specific times, focal staining that colocalizes with DAPI dense pericentric heterochromatin. (C) Immuno-RNA FISH for Dnmt3b (green) and Xist RNA (red) in XX ES cells and following differentiation for the indicated times. Dnmt3b foci adjacent to Xist RNA domains, likely corresponding to the X chromosome centromere, were seen at day 3 (short arrows). At later stages, Xist RNA domains stain negatively for Dnmt3b as shown in expanded boxes (long arrows). (D) Sequenom EpiTyper analysis of CGIs along the length of the X chromosome (indicated in schematic) in WT, Dnmt3a, Dnmt3b, and Dnmt3L mutant XX E9.5 embryos as indicated. Average values shown were determined from a minimum of two embryos of each genotype. Error bars indicate the SD between values for individual embryos. See also Table S1.

We went on to assess how the timing of Xi CGI methylation in differentiating XX ES cells relates to expression and localization of the de novo methyltransferases Dnmt3a and Dnmt3b. Western blot analysis revealed that the levels of both proteins first increased, peaking within 1–2 days of differentiation (Figure 5A). By day 4, Dnmt3a levels had decreased significantly and were considerably depleted by day 7. Dnmt3b levels remained at a constant level until day 5 and then dropped to below predifferentiation levels on day 7. It should be noted that there is evidence that levels of Dnmt3a/3b are reduced in undifferentiated XX ES cells as a result of both X chromosomes being active (Zvetkova et al., 2005).

Immunofluorescence (IF) experiments demonstrated general nuclear localization of Dnmt3a/b (Figure 5B). Localization to pericentric heterochromatin domains was also observed, and this varied through differentiation as described previously in XY ES cells (Bachman et al., 2001). Immuno-RNA fluorescence in situ hybridization (FISH) analysis detecting Xist RNA together with Dnmt3b (Figure 5C) or Dnmt3a (data not shown) demonstrated that no detectable enrichment over Xi domains occurs during XX ES cell differentiation. These results suggest that neither Dnmt3a nor Dnmt3b is actively targeted to Xi.

To further investigate the role of de novo methyltransferases in Xi CGI methylation, we carried out Sequenom EpiTyper analysis to determine the methylation levels of Xi CGIs assigned to different dynamic categories in XX embryos deleted for *Dnmt3a*, *Dnmt3b*, or *Dnmt3*L, encoding a Dnmt3a/b accessory factor (Figure 5D; Table S1) (Hermann et al., 2004). *Dnmt3b*-deleted embryos show developmental defects at E11.5 onward (Okano et al., 1999), and we therefore analyzed Xi CGI methylation in XX embryos at E9.5, when development appears normal. Deletion of *Dnmt3a* and *Dnmt3L* had no noticeable effect on Xi CGI methylation, but in *Dnmt3b* mutants, CGI methylation was reduced to levels similar to those seen in XY cells. Moreover, methylation was reduced to intermediate levels in *Dnmt3b* heterozygote XX embryos, demonstrating a dose-dependent requirement for *Dnmt3*b in Xi CGI methylation. Importantly, methylation of CGIs representing the different dynamic classes was affected similarly in mutant embryos, indicating that the use of distinct de novo methyltransferases does not determine Xi CGI methylation rates.

### CGI Methylation Dynamics Are Linked to Smchd1 Recruitment to Xi

**Figure 6:**
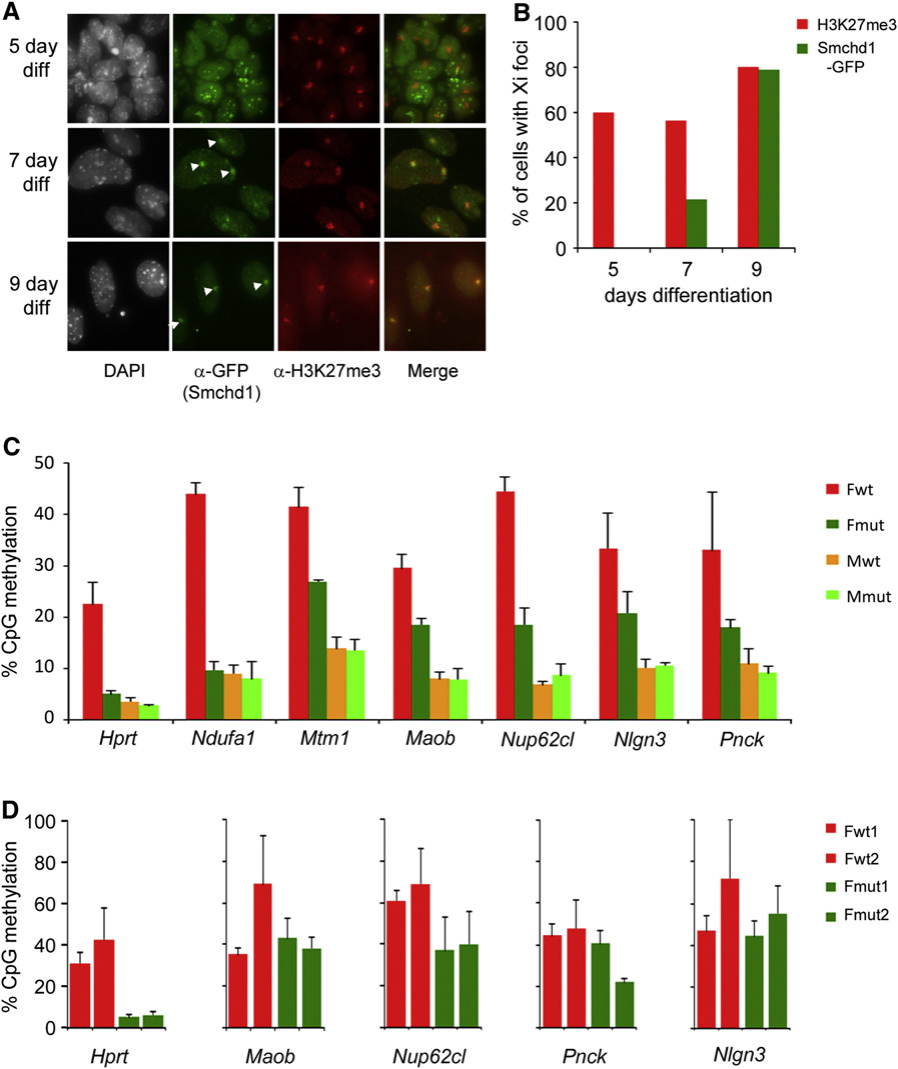
Smchd1-Dependent and -Independent DNA Methylation of Xi CGIs (A) Examples of IF analysis illustrating staining for GFP-tagged Smchd1 (green) and H3K27me3 (red) in XX ES cells lines differentiated (diff) for the times indicated. Xi domains, detected as strong H3K27me3 foci, that are also enriched for GFP-Smchd1 are indicated with arrows. DNA is counterstained with DAPI. (B) Scoring of IF data illustrates the percentage of cells with Xi domains detected using antibody to H3K27me3 and Smchd1-GFP at 5 days (n = 230), 7 days (n = 195), and 9 days (n = 166) of differentiation. (C) Histogram plots showing average CpG methylation levels at selected CGIs that are either unmethylated (*Hprt* and *Ndufa1*) or methylated (*Mtm1*, *Maob*, *Nup62cl*, *Nlgn3*, and *Pnck)* in differentiated XX ES cells. Sequenom EpiTyper analysis of bisulfite-treated DNA was carried out on XX (Fwt), *Smchd1*^*−/−*^ XX (Fmut), XY WT (Mwt), and XY *Smchd1*^*−/−*^ (Mmut) E10.5 embryos. Error bars indicate the SD of average CpG methylation in individual embryos (n = 3). (D) Histogram plots showing average CpG methylation levels at *Hprt*, *Maob*, *Nup62cl*, *Nlgn3*, and *Pnck* CGIs determined by Sequenom EpiTyper analysis of bisulfite-treated DNA from two independent XX MEF cell lines derived from E10.5 WT (Fwt1 and Fwt2) and *Smchd1*^*−/−*^ (Fmut1 and Fmut2) embryos. Error bars indicate the SD of average CpG methylation levels from independent determinations (n = 3). See also Figure S1 and Table S1.

The chromosomal protein Smchd1 plays an important role in acquisition of Xi CGI methylation (Blewitt et al., 2008; Blewitt et al., 2005). To investigate whether Smchd1 is important in defining methylation dynamics, we first analyzed the timing of Smchd1 localization to Xi using XX ES cell lines carrying a GFP-tagged Smchd1 BAC transgene. Western blot analysis demonstrated that the transgene encoded Smchd1 protein is expressed at a level similar to or lower than that observed for endogenous protein (Figure S1). We carried out immunofluorescence analysis to determine the enrichment of Smchd1-GFP on Xi interphase territories as defined by staining for H3K27me3, a marker for the inactive X chromosome that is established concurrently with the onset of Xist RNA expression (Plath et al., 2003; Silva et al., 2003). Smchd1 enrichment on Xi was almost undetectable at day 5 of differentiation but then increased rapidly between days 7 and 9, when it was detected in association with essentially all Xi territories (Figures 6A and 6B).

The fact that fast-methylating CGIs accumulate methylation prior to day 7 of differentiation indicates that methylation of these sites may be Smchd1 independent. To further investigate this, we carried out Sequenom EpiTyper analysis to determine the methylation levels of CGIs with either fast or slow DNA methylation dynamics in E10.5 *Smchd1* null (*Smchd1*^*−/−*^) XX embryos and wild-type (WT) controls (Figure 6C and Table S1). Slow-methylating CGIs (*Hprt* and *Ndufa1* loci) in XX *Smchd1*^*−/−*^ embryos have methylation levels similar to those in male embryos (Xa only), indicating complete hypomethylation, consistent with previous data (Blewitt et al., 2008). However, fast-methylating CGIs (*Mtm1*, *Maob*, *Nup62cl*, *Nlgn3*, and *Pnck*) were all significantly methylated in *Smchd1*^*−/−*^ XX embryos, albeit not to the maximal level seen in XX control embryos. Analysis of a subset of these CGIs in XX mouse embryo fibroblast (MEF) cell lines derived from *Smchd1*^*−/−*^ embryos confirmed that significant methylation occurs only in CGIs with fast methylation dynamics (Figure 6D).

**Figure 7:**
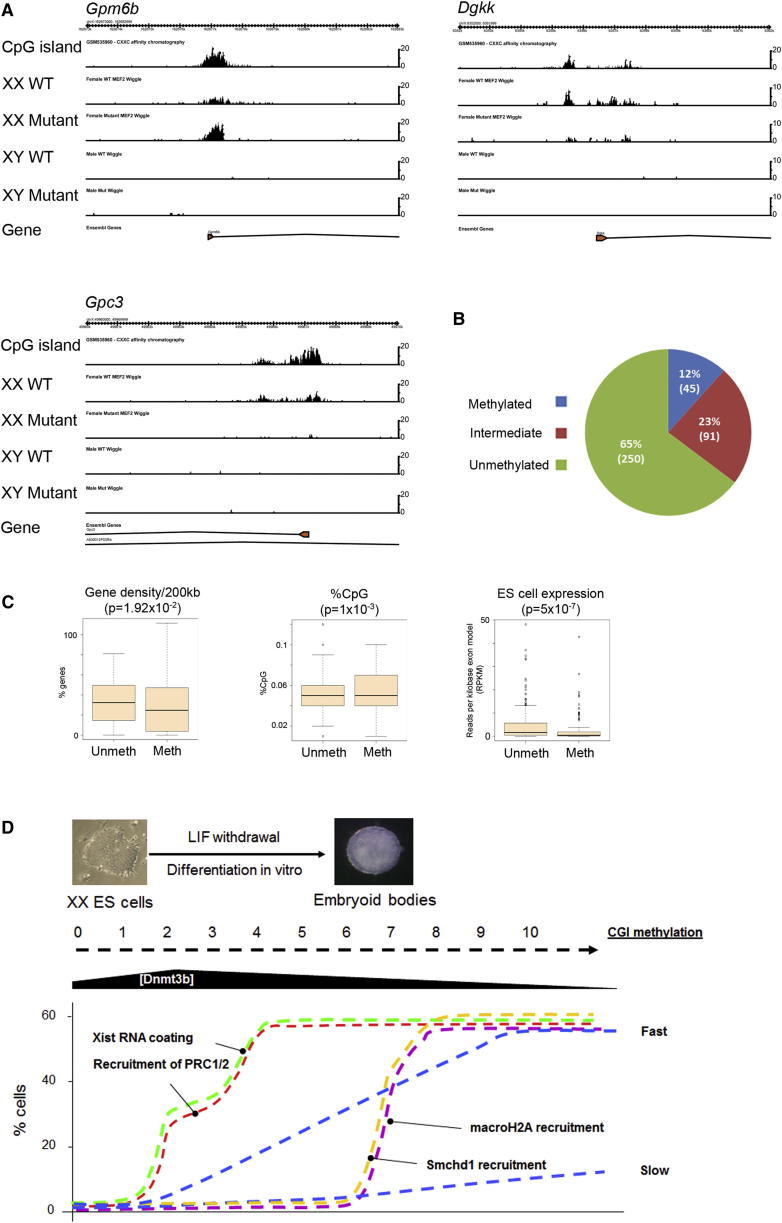
Chromosome-wide Analysis of Smchd1-Independent Xi CGI Methylation (A) Browser screenshots showing MBD-seq data over 10 kb regions, illustrating examples of patterns of methylation in CGIs associated with X-linked genes as indicated. MBD-seq tracks show data for WT and *Smchd1*^*−/−*^ (mutant) XX and XY MEF cell lines. The y axis indicates number of reads. (B) Pie chart illustrating the proportion of Xi CGIs with high, intermediate, and low levels of CGI methylation (as defined in the text) in *Smchd1*^*−/−*^ XX MEFs. (C) Illustrative examples of significant differences in gene density, % CpG, and ES cell expression level of associated genes from a comparison of unmethylated (unmeth) and methylated (meth) CGIs in *Smchd1*^*−/−*^ XX MEFs. (D) Schematic illustrating the relation of CGI methylation kinetics, Smchd1 recruitment, and Dnmt3b expression in differentiating XX ES cells. XX ES cell differentiation to form embryoid bodies is triggered by withdrawal of LIF from the culture medium. Methylation kinetics of fast and slow CGIs are represented by the dashed blue line. Upregulation of Xist RNA (green dashed line) occurs in a large proportion of cells between days 1 and 4 of differentiation, and this is closely followed by early markers of X inactivation, such as recruitment of Polycomb repressors PRC1/2 (red dashed line). Fast and intermediate methylating CGIs gain methylation progressively during the period in which Dnmt3b levels ([Dnmt3b]) remain high, and this occurs independently of Smchd1 enrichment on Xi at days 7–9 (yellow dashed line). Smchd1 enrichment occurs concurrently with recruitment of the variant histone macroH2A (purple dashed line). See also Figure S2 and Tables S2, and S5.

We extended these findings by using MBD-seq to assess Xi CGI methylation chromosome wide on an *Smchd1*^*−/−*^ background (Figures 7A–7C). To obtain sufficient material for MBD-seq, we analyzed DNA from an *Smchd*1^−/−^ XX MEF cell line, as described above. We found that although many Xi CGIs are unmethylated in *Smchd1*^−/−^ XX MEFs, a significant proportion have either high or intermediate levels of methylation. Examples of a highly methylated CGI (*Gpm6b*), an intermediately methylated CGI (*Dgkk*), and an unmethylated CGI (*Gpc3*) are shown in Figure 7A.

Using the set of CGIs that we assigned to different methylation dynamic classes based on our experiments in XX ES cells (Table S2), we categorized the CGIs in *Smchd1*^*−/−*^ XX MEFs into three groups: methylated, intermediate, and unmethylated (Figure 7B; see Supplemental Experimental Procedures). We found a highly significant overlap between class A (fast-methylating) CGIs and the combined category of methylated and intermediate CGIs in *Smchd1*^*−/−*^ XX MEFs (Pearson’s chi-square test, p = 7.43 × 10^−5^), and similarly between class C (slow-methylating) CGIs and those that remain unmethylated in *Smchd1*^*−/−*^ MEFs (Pearson’s chi-square test, p = 1.35 × 10^−5^). In comparison, overlaps with the dynamic classes B (day 10 only) and D (other) were not significant (Table S4).

To substantiate the data linking fast methylation dynamics and Smchd1 independence, we analyzed several CGIs that had been categorized as methylated in *Smchd1*^*−/−*^ XX MEFs for methylation dynamics in differentiating XX ES cells using Sequenom EpiTyper (Figure S2; Table S1). Six of a total of nine CGIs that showed Smchd1-independent methylation in MEFs had significant levels of CGI methylation in differentiated XX ES cells at days 7 and 10.

We went on to compare the characteristics of Xi CGIs that are Smchd1 dependent (unmethylated class) or Smchd1 independent (combining methylated and intermediate classes), using the same parameters as described above for analysis of methylation dynamic classes. The complete data set is given in Table S5, and selected examples are shown in Figure 7C. Consistent with the observed correlation of dynamic classes and dependence on Smchd1, similar parameters were found to discriminate Smchd1-dependent and -independent CGI classes. Specifically, Smchd1-dependent CGIs lie within regions of relatively high gene density and have reduced CpG density/GC content, and associated genes have relatively high expression levels in ES cells. Taken together, our results delineate parallel Smchd1-dependent and -independent pathways for CGI methylation in X chromosome inactivation.

## Discussion

In this study, we have shown that CGI methylation in X inactivation proceeds along two parallel pathways. Thus, a subset of CGIs are methylated at a relatively fast rate following the onset of X inactivation, in many cases independently of the chromosomal protein Smchd1. Methylation of other CGIs proceeds relatively slowly and requires Smchd1. Both pathways require the de novo methyltransferase Dnmt3b, but not Dnmt3a or Dnmt3L. Further analyses indicated that the methylation dynamics and Smchd1 dependence may be linked to sequence composition and the immediate chromosomal environment of the CGIs, as well as the expression level of the CGI-associated transcript in ES cells.

### CGI Methylation in X Inactivation

Our analysis of Dnmt3 mutants demonstrates a specific role for Dnmt3b in Xi CGI methylation and suggests that Dnmt3a and Dnmt3L are dispensable. We did not observe enrichment of Dnmt3a/b over Xi territories. This may indicate either low-level active recruitment of Dnmt3b or passive recruitment through recognition of Xi CGI chromatin. In either instance, the low levels of Dnmt3b enrichment on Xi are consistent with the gradual progressive nature of methylation at the majority of Xi CGIs.

A primary role for Dnmt3b in Xi CGI methylation is consistent with a previous study that showed reduced Xi CGI methylation in lymphoblastoid cell lines from females with immunodeficiency-centromeric instability-facial anomalies (ICF) syndrome, which is caused by mutations in the human *DNMT3B* gene (Hansen et al., 2000; Hansen et al., 1999). A role for Dnmt3a or Dnmt3L has not previously been tested. It is interesting to note that a recent study demonstrated a specific requirement for Dnmt3b in acquisition of CpG methylation in non-Xi-associated CGIs during normal development (Borgel et al., 2010). These findings, together with our results, suggest that some specific characteristics of the Dnmt3b enzyme are required to overcome the barriers that in normal circumstances protect CGIs from de novo DNA methylation.

XX ES cells have been used extensively to model chromatin changes on Xi in response to Xist RNA coating. These studies have revealed that characteristic Xi chromatin modifications generally occur during one of two phases: (1) immediately following the onset of Xist RNA expression, for example, the Xist RNA-dependent recruitment of Polycomb repressor complexes and associated histone modifications (de Napoles et al., 2004; Silva et al., 2003), or (2) several days after the onset of differentiation, for example, enrichment of histone macroH2A (Mermoud et al., 1999) and, as demonstrated more recently, recruitment of Ash2l and SAF-A (Pullirsch et al., 2010). Our results demonstrate that DNA methylation of CGIs on Xi follows a distinct pattern, in most cases accumulating at a slow rate throughout the differentiation process or during normal embryo development in vivo, and in a significant subset of CGIs accumulating at a fast rate immediately following the onset of Xist RNA expression.

A summary of these results is shown in Figure 7D. Set against the slow, progressive accumulation of CGI methylation, we find that the recruitment of Smchd1 to Xi, on which methylation of many CGIs is dependent, occurs synchronously at a defined time point within the late Xi chromatin modification phase. The kinetics of Smchd1 recruitment closely mirrors that of histone macroH2A (Mermoud et al., 1999). Presumably, Smchd1 either recruits Dnmt3b or modifies Xi heterochromatin in such a way as to allow Dnmt3b to access and methylate CGIs. Smchd1 recruitment occurs at a time when levels of Dnmt3a/b have subsided, and this could explain why CGI methylation normally proceeds slowly in differentiating XX ES cells. It is also possible that slow methylation kinetics occur because Smchd1 only partially overrides CGI protection. It should be noted that we do not know at this point whether Smchd1 enables CGI methylation directly or by bringing about other chromatin changes associated with late-phase Xi heterochromatin, such as macroH2A, Ash2l, or SAF-A enrichment. In future studies, it will be interesting to determine the interdependence of these late-phase Xi modifications.

Methylation in Smchd1-independent CGIs appears to correlate with more rapid methylation kinetics in differentiating XX ES cells, possibly because these CGIs can acquire methylation during the stage when Dnmt3b levels are relatively high (Figure 7D). Our results regarding methylation rates in vivo during normal development indicate that similar considerations are likely to apply. At present, detailed knowledge concerning the timing of Smchd1 recruitment to Xi in normal development and in relation to CGI methylation kinetics is lacking.

Although in *Smchd1*^−/−^ embryos we observed significant CGI methylation in CGIs that showed fast methylation kinetics in differentiating XX ES cells, the overall level of methylation was lower than in the WT. This observation indicates that although Smchd1 is not required to methylate these CGIs, it does contribute to the ultimate acquisition of WT methylation levels.

### Global Methylation Patterns on Xa and Xi

We observed that acquisition of CGI methylation on Xi was accompanied in most cases by loss of intergenic and intronic CpG methylation relative to Xa. This observation is consistent with a number of reports that have indicated that the overall levels of methylation on Xi are reduced relative to Xa and autosomes (Bernardino-Sgherri et al., 2002; Bernardino et al., 2000; Hellman and Chess, 2007; Viegas-Pequignot et al., 1988; Weber et al., 2005), and moreover that Xi is hypomethylated at intronic CpGs (Hellman and Chess, 2007; Lock et al., 1986).

Two models could account for the hypomethylation of intergenic/intronic sequences on Xi: (1) sites are actively methylated on Xa but not on Xi, and (2) methylation is inefficiently maintained on Xi. Because we and others (Hellman and Chess, 2007; Zvetkova et al., 2005) have shown that nonisland CpGs on the X chromosome are hypermethylated in ES cells, it would appear that methylation is lost on Xi following X inactivation. The mechanistic basis for the redistribution of CpG methylation is unclear. One possibility is that Dnmt3a/b activity is titrated away from intronic/intergenic sites as a result of ongoing CGI methylation. Alternatively, a feature of Xi heterochromatin may afford protection from methylation at these sites. It was recently shown that L1 repeat sequences are transcribed from both Xa and Xi at the onset of X inactivation, and that Xi L1 transcription is retained through differentiation (Chow et al., 2010). This could conceivably be linked to the loss of non-CGI methylation.

### Determinants of CGI Methylation

The existence of an autonomous pathway for CGI methylation is supported by the observation that a significant proportion of Xi CGIs acquire methylation in Smchd1 mutant embryos/cell lines. This defines a parallel pathway for Xi CGI methylation. As noted above, the Smchd1-independent pathway correlates with more rapid CGI methylation kinetics during development. Previous studies have indicated that the kinetics of gene silencing vary across the X chromosome (Lin et al., 2007), and it is possible that variable CGI methylation kinetics mirror this pattern. We consider this to be unlikely because variable timing of gene silencing in X inactivation correlates with location relative to *Xist* (Lin et al., 2007), whereas CGI methylation dynamics, in general, do not.

Our analyses indicate that Xi CGI methylation dynamics and dependence on Smchd1 are linked to inherent features of CGIs, notably CpG density/GC content, and also to local gene density and expression levels of associated genes in ES cells. To generalize these findings, it appears that CGIs that exhibit fast methylation kinetics and/or Smchd1 independence have a relatively high CpG density, occur in regions with reduced gene density, and are expressed at relatively low levels in ES cells. There is little or no correlation between these features and those previously associated with CGIs that have a propensity to acquire methylation during normal ontogeny (Bock et al., 2006; Borgel et al., 2010; Illingworth et al., 2008). For example, reduced rather than elevated CpG density was previously correlated with the propensity of CGIs to acquire methylation during normal ontogeny (Illingworth et al., 2010; Weber et al., 2007).

Because a number of different parameters appear to be linked to the different Xi CGI methylation pathways, it is difficult to assign relative importance. Nevertheless, the relationship with gene expression levels in ES cells is intriguing in light of evidence suggesting a role for H3K4 methylation, a transcription-associated histone modification, in blocking de novo methyltransferase activity (Ooi et al., 2007; Zhang et al., 2010). Specifically, expression levels of X-linked genes in ES cells can be inferred to approximate their status at the time in development when X inactivation is initiated. Assuming that CGIs that are silent or poorly expressed in ES cells have relatively low levels of H3K4 methylation, one could expect them in turn to exhibit an enhanced rate of acquisition of methylation and Smchd1 independence. Consistent with this idea, we observed that X-linked promoters that are targets for the Polycomb repressor protein Ring1B are more likely to show fast methylation kinetics and/or Smchd1-independent methylation. This may also link to findings indicating that a high proportion of methylated CGIs in cancer cell lines (Ohm et al., 2007; Schlesinger et al., 2007; Widschwendter et al., 2007) and neuronal cells (Mohn et al., 2008) are targets of PcG repression in early development.

What are the implications of this work for our wider understanding of CGI methylation? Because CGIs on Xa generally remain unmethylated, it is unlikely that X-linked CGIs evolved specific sequence features that render them more susceptible to methylation. The X inactivation process must therefore override mechanisms that normally protect CGIs from Dnmt3a/b activity. Smchd1 is clearly a major determinant of this, either directly or indirectly, and it will be interesting in the future to determine whether it has a role in other situations where CGI methylation occurs. Fast-methylating CGIs, however, demonstrate a second pathway that we speculate depends on the chromatin configuration/histone modification state immediately prior to the onset of X inactivation. Further studies on these pathways should help to elucidate the mechanism by which CGIs are normally protected from the DNA methylation machinery.

## Experimental Procedures

### DNA Methylation Assays

DNA methylation was assayed by MSRE and bisulfite-based analysis. PCR products from bisulfite-treated DNA were analyzed by direct sequencing or by using the Sequenom EpiTyper assay. Further details are provided in Supplemental Experimental Procedures.

### Protein Detection

Western blots were performed using antibodies specific for Dnmt3a and Dnmt3b (Alexis), Lamin B (Santa Cruz), Smchd1 (Abcam), and GFP (Roche). Sample preparation, IF, and immuno-RNA FISH were carried out essentially as described previously (de Napoles et al., 2004; Mak et al., 2002; Silva et al., 2003) using primary antibodies to detect Dnmt3a and Dnmt3b (Alexis), H3K27me3 (Millipore), and GFP (Roche). Further details are provided in Supplemental Experimental Procedures.

### High-Throughput Analysis of DNA Methylation

Purification of methylated DNA using the MBD column was adapted from a previously described method (Cross, 2002). Further details are provided in Supplemental Experimental Procedures. MBD-seq was obtained by the single-end method using Illumina Genome analyzer II as detailed in Supplemental Experimental Procedures. Tags were mapped using bowtie (Langmead et al., 2009) excluding nonunique mappings (the -m 1 option). The GEO accession number for MBD-seq data is GSE37333.

Following alignment to the mouse genome (mm9), data were visualized on GBrowse (Stein et al., 2002). Further analysis of the data used SeqMonk (http://www.bioinformatics.bbsrc.ac.uk/projects/seqmonk/). Full details are provided in Supplemental Experimental Procedures.

To define CGI methylation dynamics classes, probe counts from Seqmonk analysis were calculated as % of XX WT MEF and then filtered as follows: class A: methylation at day 7 of differentiation ≥ 10%, and methylation at day 10 of differentiation > 75%; class B: methylation at day 7 of differentiation < 10%, and methylation at day 10 ≥ 70%; class C: methylation at day 10 < 10%; class D: all CGIs not allocated to classes A–C. For comparison of methylation levels in WT and Smchd1 null XX MEFs, CGIs were classified relative to methylation levels in XX WT MEF as follows: methylated in Smchd1 null XX MEF, ≥75% methylation; intermediate methylation in Smchd1 null XX MEF, ≥25% methylation and <75% methylation; and unmethylated in Smchd1 null XX MEF, <25% methylation.

### Statistics

Pairwise comparisons were performed for methylation dynamics classes A–D and then for methylated and unmethylated in *Smchd1*^*−/−*^ MEF CGI classes assessing 21 variables (Supplemental Experimental Procedures) using the Wilcoxon test statistic.

For each one of the 21 variables, we tested against the null hypothesis of equality of distribution between two classes (50 fast-methylating CGIs, 44 CGIs methylated at day 10 of differentiation only, 156 slow-methylating CGIs, and 136 CGIs that did not fall within any of defined classes). We considered all pairwise comparisons (class A versus class B, class A versus class C, class A versus class D, class B versus class C, class B versus class D, and class C versus class D), as well as classes A+B versus class C and classes A+B versus class D. The Wilcoxon test statistic was used to detect a shift in location.

For each test, three p values were computed. The first was obtained using the asymptotic null distribution of the test statistic, the second was calculated using Monte-Carlo resampling (approximate p value), and the third was computed using the shift algorithm described in Streitberg and Rohmel (1986) (exact p value) and implemented in the coin package in R software. The exact p value does not rely on the asymptotic assumptions of the test performed and is therefore considered more accurate. For each variable, in order to control the family-wise error rate, we apply a Bonferroni correction and test each individual hypothesis at a significance level of 0.05/8 (0.00625). However, such a correction can be overly stringent, and significance is also investigated for p values < 0.05.

The same statistical analysis was performed for the pairwise comparisons between the classes of methylated (45 CGIs), intermediate (91 CGIs), and unmethylated (250 CGIs) CGIs across all 21 target variables. We considered the following pairwise comparisons: methylated versus unmethylated, methylated versus intermediate methylated, unmethylated versus intermediate methylated, and methylated versus intermediated methylated and unmethylated combined. In this case, the strict Bonferroni-adjusted significance level of the p value is 0.05/4 (0.0125).

Finally, a Pearson’s chi-square test of homogeneity was performed for each class of CGIs (classes A–D) to test against the null hypothesis of equality of distribution between the observed frequencies and those expected by chance (as obtained by classifying the CGIs as methylated or unmethylated in Smchd1 null XX MEFs). For each class, a p value was computed from the asymptotic chi-square distribution of the test statistic.

## Supplemental Information

Document S1. Figures S1 and S2, Table S4, and Supplemental Experimental Procedures
