# Supplementary material for: Smchd1-Dependent and -Independent Pathways Determine Developmental Dynamics of CpG Island Methylation on the Inactive X Chromosome
Source: Dev Cell. 2012 Aug 14;23(2):265–79. doi: 10.1016/j.devcel.2012.06.011 (PMC3437444; doi:10.1016/j.devcel.2012.06.011)
Supplement: Document S1. Figures S1 and S2, Table S4, and Supplemental Experimental Procedures [file mmc1.pdf]

Developmental Cell, Volume *23*

Supplemental Information

Smchd1-Dependent and -Independent Pathways Determine Developmental Dynamics of CpG Island Methylation on the Inactive X Chromosome

Anne-Valerie Gendrel, Anwyn Apedaile, Heather Coker, Ausma Termanis, Ilona Zvetkova, Jonathan Godwin, Y. Amy Tang, Derek Huntley, Giovanni Montana, Steven Taylor, Eleni Giannoulatou, Edith Heard, Irina Stancheva, and Neil Brockdorff

Inventory of Supplementary Materials

Figure S1. Endogenous Smchd1 and Smchd1-GFP are expressed at similar levels in ES cells. Related to Figure 6.

Figure S2. CGI methylated in *Smchd1* null XX MEFs show significant levels of methylation in differentiated XX ES cells. Related to Figure 7.

Table S1. Sequenom Epityper raw data for Figures 1B, 3C, 5D, 6C, 6D and S2. Related to Figures 1, 3, 5, 6, and S2 (Legend in Supplementary Materials document, Table in separate file)

Table S2. Methylated X-linked CGIs analysed following MBD-seq. Related to Figures 4 and 7. (Legend in Supplementary Materials document, Table in separate file)

Table S3. Parameters linked to classes of Xi CGI with different methylation dynamics in differentiating XX ES cells. Related to Figure 4. (Legend in Supplementary Materials document, Table in separate file)

Table S4. Statistical analysis of the overlap comparing methylation dynamic classes with CGIs methylated or unmethylated in *Smchd1* null XX MEFs.

Table S5. Parameters linked to Xi CGI methylation in *Smchd1* null MEF cell lines. Related to Figure 7. (Legend in Supplementary Materials document, Table in separate file)

Supplemental Experimental procedures

Supplemental references

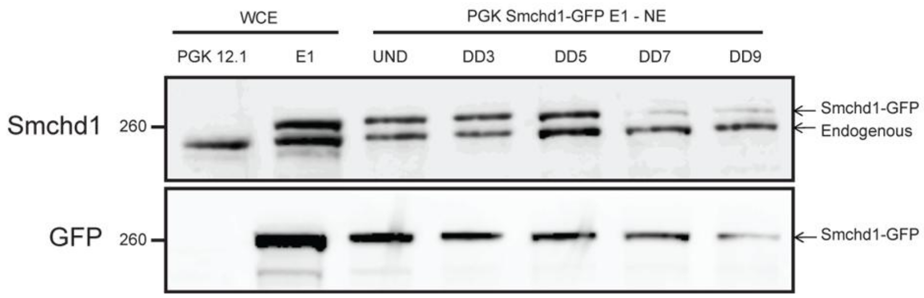

Figure S1. Endogenous Smchd1 and Smchd1-GFP are expressed at similar levels in ES cells. Related to Figure 6.

Whole-cell (WCE, lane 1 & 2) and nuclear extracts (NE, lane 3 to 7) were prepared from untransfected ES cells (ES) and from an ES cell line stably expressing an Smchd1-GFP BAC transgene (ES Smchd1-GFP). ES Smchd1-GFP cells were differentiated for 0 (UND), 3, 5, 7 and 9 days (DD) as indicated. Comparison of expression levels between endogenous Smchd1 and Smchd1-GFP was analysed by Western-blot using anti-Smchd1 and anti-GFP antibodies.

.

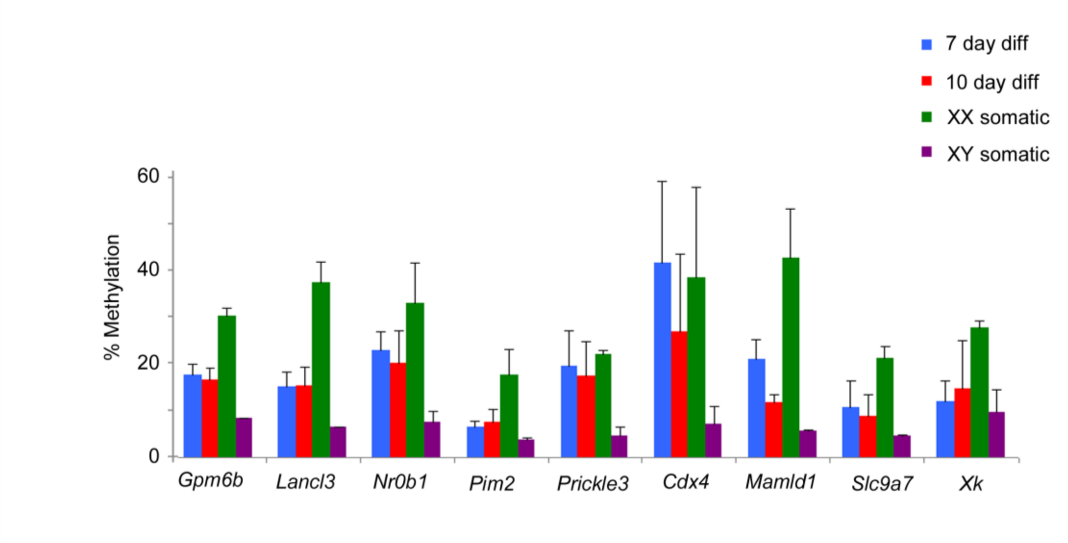

Figure S2. CGI methylated in *Smchd1* null XX MEFs show significant levels of methylation in differentiated XX ES cells. Related to Figure 7.

Methylation patterns of selected CGI methylated in *Smchd1* null XX MEFs were analysed by Sequenom EpiTyper in XX ES cells differentiated as indicated and in XX and XY somatic tissues (adult spleen). Average methylation levels were calculated for each fragment from a minimum of two samples; Error bars indicate standard deviation between values from independent samples.

Supplemental Tables

Table S1. Sequenom Epityper raw data for Figures 1B, 3C, 5D, 6C, 6D and S2. Related to Figures 1, 3, 5, 6, and S2

(see accompanying Excel file)

Each sheet lists data used in different figure sections as indicated. For each gene (Sample ID) CpG site analysed is shown with data from two to four biological replicates of different cell types/tissues. Additional columns show the average and standard deviation (stdev) values for each set of replicate values. Additional rows show average value across all CpG sites analysed for a given gene.

Table S2. Methylated X-linked CGIs analysed following MBD-seq. Related to Figures 4 and 7.

(see accompanying Excel file)

Table shows start and end position of 387 Xi CGIs, nearest associated gene (feature), other characteristics (note), assignment to methylation dynamic class, and methylation status in Smchd1 null XX MEFs.

Table S3. Parameters linked to classes of Xi CGI with different methylation dynamics in differentiating XX ES cells. Related to Figure 4

(see accompanying Excel file)

Each sheet lists results from pairwise comparisons between each dynamic class of CGI as described in Figure 4 and the accompanying text; Class A (Fast), Class B (day 10 only), Class C (slow), and Class D (other). Additionally, comparisons were made using a combination of Class A and Class B (combined) with both Class C and Class D. Tables report on the significance of differences observed and the distribution of values is shown in the boxplots. Parameters analysed for each class are explained in supplemental experimental procedures.

Table S4. Statistical analysis of the overlap comparing methylation dynamic classes with CGIs methylated or unmethylated in *Smchd1* null XX MEFs.

Total 386 CGI	Class A (50)	Class B (44)	Class C (156)	Class D (136)
All meth (136)	31	19	29	57
Unmeth (250)	19	25	127	79
P-value (Asymptotic)	7.428e-05	0.2697	1.351e-05	0.103

Overlap of Xi CGIs belonging to classes A-D (Figure 4) and Xi CGIs that are methylated either independently of Smchd1 (All meth) or dependent on Smchd1 (Unmeth).

Table S5. Parameters linked to Xi CGI methylation in *Smchd1* null MEF cell lines. Related to Figure 7.

(see accompanying Excel file)

Results from pairwise comparison of Xi CGIs unmethylated in *Smchd1* null XX MEFs and CGIs with either high or intermediate levels of methylation as described in Figure 7 and the accompanying text; Tables report on the significance of differences observed and the distribution of values is shown in the boxplots. Parameters analysed for each class are explained in supplemental experimental procedures.

Supplemental Experimental Procedures

Cell lines and mouse strains

XX PGK12.1 ES cells were maintained and differentiated as described previously (Norris et al., 1994; Penny et al., 1996). ES cell lines stably expressing GFP tagged-Smchd1 were derived in house by transfecting PGK12.1 cells with a retrofitted Smchd1 BAC clone (kind gift of Mitocheck (http://www.mitocheck.org/)). Further details are available on request. Xi specific intergenic/intronic methylation analysis was carried out using T16H/Cast fibroblast cells (Gomez and Brockdorff, 2004). For somatic methylation analysis DNA was prepared from cell lines and (C57Bl6 x CBA) F1 somatic tissues (kidney or spleen) using standard methods. Embryos were obtained from timed matings of (C57Bl6 x CBA) F1 mice or from Smchd1 or Dnmt3L heterozygote intercrosses. Dnmt3a and Dnmt3b mutant embryos were obtained from crosses with conditional floxed alleles and the oocyte specific CRE recombinase, ZP3-CRE (Lewandoski et al., 1997). Embryos were sexed and/or genotyped as described previously (Blewitt et al., 2008; Bourc'his et al., 2001; Kaneda et al., 2004). Mouse embryonic fibroblasts (wild-type and *Smchd1* null male and female) were obtained from E10.5 embryos. Permanent cell lines were established by passaging primary cells through growth crisis.

DNA methylation assays

For Southern-blot assays, DNA was digested with appropriate restriction enzymes in accordance with the manufacturers' recommendations and then separated by electrophoresis on 1% agarose gels, Southern blotted and hybridised with radiolabelled probes using standard procedures. Details of Southern blot probes are available on request.

For conventional bisulfite sequencing we processed 1ug DNA per sample using a bisulfite assay kit (Chemicon International) in accordance with the manufacturer's protocols. The bisulfite conversion was carried out at 50°C for 8-16 hours and 2µl of sample was used for each PCR reaction. After PCR amplification (nested in some cases), samples were cloned into pCR-4-TOPO (Invitrogen) and sequenced. Results were analysed using BiQAnalyser, QUMA, ChromasLite and Lasergene. Primer sequences are listed in "Primers used for bisulfite sequencing and Sequenom Epityper analysis" section below. Primers were designed in the CGI of *Mtm1*, *Hprt* and *Pdk3* and overlapping polymorphisms between *M.castaneus* and *M.domesticus* strains. For the following, primers were designed in intergenic or intronic regions. Mtm1-US is an intergenic region located 10kb upstream of *Mtm1* and has 4 CpGs and 2 polymorphisms within 671bp. Abcd1-DS is an intragenic region located within the second intron of *Abcd1*, around 25kb downstream of the start site, and has 6 CpGs and 6 polymorphisms within 368bp. Mospd1-US is an intergenic region located 14kb downstream of *Mospd1* and contains 3 CpGs and 1 polymorphism within 400bp. Hprt-DS is an intragenic region located in intron 5,26kb downstream of the start of *Hprt* and contains 5 CpGs within 380bp, but has no polymorphisms.

For Sequenom EpiTyper analysis genomic DNA from XX and XY somatic tissues and XX ES cells (day-7 and day-10 differentiated), embryos (E6.5 and E10.5), and MEF cells were bisulfite treated using the EZ DNA Methylation kit (Zymo Research, Cambridge

BioSciences). Approximately 2ug of genomic DNA per sample (except for E6.5 where all DNA extracted from a single embryo was used) was modified following the manufacturer's instructions, except the conversion reaction was carried out under the following conditions: 30 seconds at 95°C and 15 minutes at 50°C for 20 cycles. Converted DNA was then purified, eluted in 100µl H_2O and 5µl was used for each PCR reaction. Regions of interest were amplified using tagged primers (see "Primers used for bisulfite sequencing and Sequenom Epityper analysis" below) and sent to Sequenom for EpiTyper methylation analysis by mass spectrometry (Ehrich et al., 2005). In most cases analysis was carried out using the T-reaction only. The primer sequences used are listed in "Primers used for bisulfite sequencing and Sequenom Epityper analysis" table below; primers for the *Rhox6*/*9*, *Ndufa1, Mcts1* and *Ube2a* CGIs have been previously described (Oda et al., 2006).

Primers used for bisulfite sequencing and Sequenom Epityper analysis.
Primer sequences, annealing temperature (Ta) and product size are listed.

Primer Pair	Foward Sequence	Reverse Sequence	Ta (°C)	Size (bp)
**BISULFITE SEQUENCING**				
**Mtm1**	TGGAGAGTTTTAGTTGTTTTTGTAAG	AAAACTAAACCATTCCTTCTCAAATC	56	426
**Hprt**	GTGGGGATGTTTTTTTAGTGAGTT	CAAACCTAAACATACCCTCTCATAC	57	280
**Hprt-snp**	AGTGATTATTTGGGAATTTTTTGG	CCTCATAAAACATCTAACCAAACTC	58	559
**Pdk3-snp**	GGAATTTGTTTAGAAGTTTAAGTAAGAGTT	AACCCCAAATAAAAATAAAACCC	54	333
**Mtm1-US**	TTTAGTATTTAGGAGGGTTGGTGAT	ACCTTTCTCAAAACCAAAATATTTCAT	58	---
**Mtm1-US nest**	TGGTGATATAAAGTTTTATTTTTAGTT	CACAATAATTACAACAAACACTCTAA	54	671
**Hprt-DS**	GGAATTTAGGGATTGAATTTAGTTT	TCAAAAAAAAAATTATCCCTCATCTA	58	---
**Hprt-DS nest**	ATTAGGTTTGTATGGTAAAATGTATTT	CAATCACACCAAAAACTATACAATAC	54	380
**Abcd1-DS**	TTAAGGAAAAGTTTGATAGGAAG	AAACCTATCTAAAAAATAACAAAAATAAC	60	---
**Abcd1-DS nest**	GAAGTTATTTAATTATTGGGGATG	CCTATCTAAAAAATAACAAAAATAAC	60	368
**Mospd1-US**	ATATTAATTGTTGGTAGGTGGTTGG	CACAACTAACAAAAATAAACACCATAAC	60	---
**Mospd1-US nest**	TTATTTTTAATTTTGGGGGTTTTAG	AAAATAAACACCATAACCTATTTTCTC	60	400
**SEQUENOM EPITYPER**				
**Hprt**	GTGGGGATGTTTTTTTAGTGAGTT	CAAACCTAAACATACCCTCTCATAC	57	280
**Mtm1-1**	GTTGGAGAGTTTTAGTTGTTTTTGTAAGG	CCTTTCCTAAACCCCAATTCCCCCATTCAA	60	746
**Mtm1-2**	TGGAGAGTTTTAGTTGTTTTTGTAAG	AAAACTAAACCATTCCTTCTCAAATC	56	426
**Rpgr**	TTTGGTATAGATTATTATTGTGTTTT	TATCCCCTCTAAATCCCTACATAAC	53	272
**Maob**	TGTTTGTTAGGTTTTAGGTTTTTTG	ATCACCCTCCCAACTAAAATACTC	55	257
**Slc25a43**	TAGAAAGAATAGGAAATGGAAGTTT	CCAAAAAAATAACTTTACACCTTACC	56	409
**Ube2a**	TTTAGGGAATTTGGTTATTTTTTTT	TACCTCTTAAAATCCCTCATAAAAC	55	381
**Ndufa1**	GATTTTTTTTAGATGGTTTTTTTT	ATCACTTTTAAACCCCCCACAAC	60	372
**Rhox6/9**	TTATTTTTTGTGAAGGAATTTGAAT	AAATCCAATCAAAACCAATTTACTC	54	488/498
**Mcts1**	GATTAAGTTGTTATTGAAAAGGAGT	AATTAAATTAAAAAAAATCCAACAC	58	178
**Ocrl**	GTAATTTGAAGAGGGATTTGTTATG	TACTACCAAAAACTAAACTCCAATC	57	383
**Gpc3**	GGTTGTAGTTTTTGGAAGAAAGA	AATAAACCCAATCTCCCAACTAAAC	58	481
**Pnck**	TGTTTGTTTTTAGTTTAGAGTGGGG	CAAAAACCCTAAAACCACTCACTTA	55	325
**Pdk3**	TTTAGGAATTGTTTGATAGATAGTGG	AACCCCAAATAAAAATAAAACCC	57	219
**Eif2s3x**	TTTGTTGGATTTTTGTGTAGGTAAG	AAAACTAAACTCTTCCTCCATCATCT	55	426
**Nlgn3**	TATAAAAGGAGTTAATGTTAGGGAT	ATAAACAACTAAAAAATAAAAAACC	55	242
**Zdhhc15**	TTTAAGATTGGAAATTGGGAGTTT	TAAACAATACAACCCCACAAATAAC	59	444
**Pgk1**	TTATTTGGGAGTTGAGAAAGTAGAG	AAAAAAAACTTCCTAACTAAAAAAAA	52	276
**Nup62cl**	TTTTTTTATTTTTTATTTTGGGTTT	AATCTAAAAACTCAATACCTTCAAC	56	383
**Phf8**	TGAAAGATTAAATGATAATTTTAAT	CTCCATTTTAAATAAAACCTAAAAC	51	291
**G6mpd**	AGTGAAAGTTATAGGTTTGTAATAG	AAATCACCCAAATAACTCAAATAAC	56	470
**Lancl3**	GTTTTAGGTTTTTTGTAGGGAGG	CCAACAAACTACCCTAATAATCATC	55	496
**Nr0b1**	GTTGTTTTTGTGGGGAGAATTATT	TCTAAACCACCCACACTCTAAATAC	58	364
**Pim2**	GTATGTATTTTGTATAAGGGGTATTTGAAA	AATCCACCCAACCTACTTATAACAA	58	458
**Prickle3**	AGGGTGATTGAGATATTATATAGGT	AAAACTTAAACCACCATATCTACAC	58	398
**Cdx4**	ATGAATGATATGATTTTTTTAGTTTT	AAACTACTAATCCCATCAACCAACTAC	58	342
**Mamld1**	TTGTTTGTGGTTATATTTTGGTTTG	AATTAAAAATCACCCCCTAACTAAC	58	375
**Slc9a7**	GGTGGTAAGTTTTTTTATGG	CTAATAACCTTACTACTCCTCTAAAC	58	418
**Xk**	GGGTTGAGTATGTTGGGAGT	CAAAACATCAAAAAAAAAAA	54	262
**10 5'tag**	AGGAAGAGAG			
**T7 3'tag**	CAGTAATACGACTCACTATAGGGAGAAGGCT			

Western-blot analysis

Whole-cell and nuclear extracts were prepared as described (Mermoud et al., 1999) and proteins were separated by SDS-PAGE (6% for Smchd1; 8% and 4-15% gradient for Dnmt3a, Dnmt3b and Lamin B) and wet-blotted in 10 mM Tris, 8.3 mM NaAc and 0.5 mM EDTA (pH 7.8). Western blotting was performed using TBS (0.9% NaCl, 100 mM Tris). Antibodies to Dnmt3a and Dnmt3b were from Alexis Biochemicals. The Dnmt3a antibody, which recognizes Dnmt3a1 and Dnmt3a2 isoforms and also cross-reacts with Dnmt3b, was diluted 1:250. The Dnmt3b antibody recognizes all isoforms of Dnmt3b and was used at a 1:250 dilution. Lamin B (Santa cruz), Smchd1 (Abcam) and GFP (Roche) antibodies were used respectively at 1:2000, 1:750 and 1:1000 dilutions. Secondary antibodies linked to horseradish peroxidase were used for ECL detection in accordance with the manufacturer's recommendations (Amersham).

IF and immuno-RNA FISH

Primary antibodies were diluted in 5% normal goat serum in 0.2% fish gelatin for anti-Dnmt3a (1:100, Alexis) and anti-Dnmt3b (1:100, Alexis), or alternatively in 1%BSA/PBS for anti-H3K27me3 (1:500, Millipore) and anti-GFP (1:50, Roche). To detect *Xist* RNA, the probe was generated using the GPT16 plasmid DNA, containing *Xist* exon 1 (Duthie et al., 1999), labelled with biotin-16-dUTP nick translation kit (Roche) according to manufacturer's instructions and detected with avidin-Texas red (AV-TR). Images were acquired on a Leica epifluorescence microscope using a CCD camera or alternatively on a Leica TCS SP1 confocal microscope.

MBD affinity purification

Purification of methylated DNA using the MBD column was adapted from the method described in (Cross, 2002). Briefly, 65mg of purified 6xHis-tagged MBD domain of MeCP2

were bound to 1ml of nickel coated Chelating Sepharose Fast Flow sepharose (GE Healthcare). MBD bound resin was packed into a 1 ml Tricorn column (GE healthcare). The column was washed using a 0.1M to 1M salt gradient three times to eliminate any bacterial DNA carried over from the MBD peptide expression and purification from *E.coli*. RNAse treated genomic DNA extracted from XX ES (day-7 and day-10 differentiated) and MEF cell lines (female wild-type and *smchd1* mutant; male wild-type and *smchd1* mutant) was sonicated to an average size of ~300bp and 20ug were loaded onto the MBD column. Unmethylated and methylated DNA fragments were eluted at low (up to 0.715M) and high (1M) NaCl concentration respectively (buffer used: 20mM HEPES pH7.5; 0.1% Triton; 10% Glycerol; 0.5mM PMSF with increasing NaCl concentration from 0.1M to 1M). The success of each fractionation was tested using actin (F: CCTAATACGGCTTTTAACACCC; R: CCTGAGGATCACTCAGAACGG) and Igf2r-DMR2 (F: TGTGGCACCCTCATGCATAG; R: AGCTATCCTGAGGGTGCGAA) PCRs as unmethylated and differentially methylated controls, respectively. Fractions most enriched in methylated DNA were pooled, concentrated and de-salted (Amicon Ultra Centrifugal filter device 30K, Millipore). The samples were precipitated and resuspended in 20ul TE buffer. Methylated DNA fractions were then analysed by high-throughput sequencing.

High-throughput-sequencing and bioinformatics

For MBD-seq DNA was end-repaired, A-tailed and adapter-ligated using a MBD-seq DNA Sample prep Kit (Illumina). A Qiagen PCR purification step was performed following each enzymatic reaction. The adapter-ligated material was then PCR amplified, using 18 cycles of PCR before size selection of 200-500 bp fragments on an agarose gel. The library was then extracted using a Qiaquick gel extraction kit and checked for concentration and integrity. DNA was sequenced using an Illumina Genome Analyser II using 51bp reads. The reads were mapped to the mouse mm9 genome using default bowtie with the –m 1 (suppress all alignments if the read maps more than once) (Langmead et al., 2009). Mapped reads for the different experiments were; 13903829 (XX WT MEF), 1937780 (7 day

differentiated XX ES cell), 2663037 (10 day differentiated XX ES cells), 29104137 (XX Smchd1 null MEF), 8903268 (XY WT MEF) and 7150486 (XY Smchd1 mutant MEF). Data were visualised in GBrowse (Stein et al., 2002). CGI were manually annotated, selecting those detected in cerebellum DNA using CAP-seq (Illingworth et al., 2010) for which there were corresponding MBD-seq peaks in XX but not XY MEFs.

We noted that some CGIs that were significantly methylated when assayed by direct methods were underrepresented following MBD chromatography, notably the *Mtm1* and *Maob* CGIs. This is because conditions were optimised to capture densely methylated CGI fragments and these are relatively small CGIs with a low CpG density.

To determine the proportion of Xi CGI showing different methylation kinetics or those that were methylated in XX Smchd1 null MEFs reads were counted and logged to base 2, then normalized to the largest (XX Smchd1 null MEF) dataset using Seqmonk (http://www.bioinformatics.bbsrc.ac.uk/projects/seqmonk/) probes designed around Ensembl gene features. The probe differences were filtered to have an average value of greater than 1.0.

Statistical Analysis

We performed pair-wise comparisons using 21 variables: Gene density (%) 50kb upstream (Up) of CGI, Gene density (%) 50kb downstream (Down) of CGI, Gene density (%) 100kb upstream and downstream of CGI combined (Combined) , Gene density (%) 100kb Up, Gene density (%) 100kb Down, Gene density (%) 200kb Combined, L1 (%) 50kb Up, L1 (%) 50kb Down, L1 (%) 100kb Combined, L1 (%) 100kb Up, L1 (%) 100kb Down, L1 (%) 100kb All, Distance from the Xist locus (bp), Overlaps Ring1B WT Peaks (fraction of CpG), CGI Length, G+C content, %CG, base per turn, stacking energy, CpG O/E ratio and expression level in undifferentitated ES cells determined by RNA-seq (Ficz et al., 2011), shown as reads per kilobase exon model (RPKM). Base per turn and stacking energy were calculated using the btwisted module within the EMBOSS package (Rice et al., 2000).

Supplemental references

Blewitt, M.E., Gendrel, A.V., Pang, Z., Sparrow, D.B., Whitelaw, N., Craig, J.M., Apedaile, A., Hilton, D.J., Dunwoodie, S.L., Brockdorff, N.*, et al.* (2008). SmcHD1, containing a structural-maintenance-of-chromosomes hinge domain, has a critical role in X inactivation. Nat Genet *40*, 663-669.

Bourc'his, D., Xu, G.L., Lin, C.S., Bollman, B., and Bestor, T.H. (2001). Dnmt3L and the establishment of maternal genomic imprints. Science *294*, 2536-2539.

Cross, S.H. (2002). Isolation of CpG islands using a methyl-CpG binding column. Methods Mol Biol *200*, 111-130.

Duthie, S.M., Nesterova, T.B., Formstone, E.J., Keohane, A.M., Turner, B.M., Zakian, S.M., and Brockdorff, N. (1999). Xist RNA exhibits a banded localization on the inactive X chromosome and is excluded from autosomal material in cis. Hum Mol Genet *8*, 195-204.

Ehrich, M., Nelson, M.R., Stanssens, P., Zabeau, M., Liloglou, T., Xinarianos, G., Cantor, C.R., Field, J.K., and van den Boom, D. (2005). Quantitative high-throughput analysis of DNA methylation patterns by base-specific cleavage and mass spectrometry. Proc Natl Acad Sci U S A *102*, 15785-15790.

Ficz, G., Branco, M.R., Seisenberger, S., Santos, F., Krueger, F., Hore, T.A., Marques, C.J., Andrews, S., and Reik, W. (2011). Dynamic regulation of 5-hydroxymethylcytosine in mouse ES cells and during differentiation. Nature *473*, 398-402.

Gomez, M., and Brockdorff, N. (2004). Heterochromatin on the inactive X chromosome delays replication timing without affecting origin usage. Proc Natl Acad Sci U S A *101*, 6923-6928.

Illingworth, R.S., Gruenewald-Schneider, U., Webb, S., Kerr, A.R., James, K.D., Turner, D.J., Smith, C., Harrison, D.J., Andrews, R., and Bird, A.P. (2010). Orphan CpG islands identify numerous conserved promoters in the mammalian genome. PLoS Genet *6*.

Kaneda, M., Okano, M., Hata, K., Sado, T., Tsujimoto, N., Li, E., and Sasaki, H. (2004). Essential role for de novo DNA methyltransferase Dnmt3a in paternal and maternal imprinting. Nature *429*, 900-903.

Langmead, B., Trapnell, C., Pop, M., and Salzberg, S.L. (2009). Ultrafast and memory-efficient alignment of short DNA sequences to the human genome. Genome Biol *10*, R25.

Lewandoski, M., Wassarman, K.M., and Martin, G.R. (1997). Zp3-cre, a transgenic mouse line for the activation or inactivation of loxP-flanked target genes specifically in the female germ line. Curr Biol *7*, 148-151.

Mermoud, J.E., Costanzi, C., Pehrson, J.R., and Brockdorff, N. (1999). Histone macroH2A1.2 relocates to the inactive X chromosome after initiation and propagation of X-inactivation. J Cell Biol *147*, 1399-1408.

Norris, D.P., Patel, D., Kay, G.F., Penny, G.D., Brockdorff, N., Sheardown, S.A., and Rastan, S. (1994). Evidence that random and imprinted Xist expression is controlled by preemptive methylation. Cell *77*, 41-51.

Oda, M., Yamagiwa, A., Yamamoto, S., Nakayama, T., Tsumura, A., Sasaki, H., Nakao, K., Li, E., and Okano, M. (2006). DNA methylation regulates long-range gene silencing of an X-linked homeobox gene cluster in a lineage-specific manner. Genes Dev *20*, 3382-3394.

Penny, G.D., Kay, G.F., Sheardown, S.A., Rastan, S., and Brockdorff, N. (1996). Requirement for Xist in X chromosome inactivation. Nature *379*, 131-137.

Rice, P., Longden, I., and Bleasby, A. (2000). EMBOSS: the European Molecular Biology Open Software Suite. Trends Genet *16*, 276-277.

Stein, L.D., Mungall, C., Shu, S., Caudy, M., Mangone, M., Day, A., Nickerson, E., Stajich, J.E., Harris, T.W., Arva, A., *et al.* (2002). The generic genome browser: a building block for a model organism system database. Genome Res *12*, 1599-1610.
